# The compliance of thromboprophylaxis affects the risk of venous thromboembolism in patients undergoing hip fracture surgery

**DOI:** 10.1186/s40064-016-2724-1

**Published:** 2016-08-18

**Authors:** Yuan Gao, Anhua Long, Zongyan Xie, Yutong Meng, Jing Tan, Houchen Lv, Licheng Zhang, Lihai Zhang, Peifu Tang

**Affiliations:** 1 Department of Orthopaedics, Chinese PLA General Hospital, 28 Fuxing Road, Haidian District, Beijing, 100853 People’s Republic of China; 2 Department of Orthopaedics, Beijing Luhe Hospital, Capital Medical University, Beijing, 101149 People’s Republic of China; 3 School of Medicine, Nankai University, Tianjing, 300071 People’s Republic of China

**Keywords:** Venous thromboembolism, Hip fracture, Compliance, Guideline adherence, Rivaroxaban

## Abstract

**Objective:**

Venous thromboembolism (VTE) is major problem after hip fracture surgery with substantial morbidity and mortality. This study aimed to assess the postoperative compliance of thromboprophylaxis in elderly patients undergoing hip fracture surgery and to confirm the correlation between compliance and VTE risk.

**Methods:**

This retrospective cohort study included consecutive elderly hip fracture patients who undergoing surgery. According to the thromboprophylaxis regimens, patients were divided into non-compliant group (<14days), poor compliant group (14–27days) and good compliant group (≥28days). The primary outcome was the incidence of symptomatic DVT, PE within 6weeks postoperatively.

**Results:**

Between 2008 and 2012, 1214 eligible patients were included in this study. 761 (64.7%) patients were non-compliant, 224 (19.0%) patients were poor compliant, and 192 (16.3%) patients were good compliant. The overall VTE rate was 7.9% (73/1177), PE rate was 0.3% (4/1177). The VTE rate in good compliant group was lowest among three groups (4.2 vs. 5.4 vs. 9.6%, P=0.013), but the PE rates showed no significant differences (0 vs. 0.9 vs. 0.3%, P=0.241). The multivariate analysis showed that non-compliance was an independent risk factor of suffering VTE undergoing hip fracture surgery.

**Conclusions:**

In this study we found fewer than 1 in 5 patients maintained compliant with thromboprophylaxis guidelines after discharge following hip surgery. This is particularly concerning because those who were non-compliant had a higher risk of VTE postoperatively compared with those who were good compliant.

## Background

Venous thromboembolism (VTE) is a leading cause of mortality among patients in hospital (Goldhaber 2012; Geerts et al. 2008). Major orthopedic surgery (include hip or knee replacement or hip fracture surgery) is associated with a high risk of VTE for a long time period (Anderson et al. 2002; Prandoni et al. 2002; Haentjens et al. 2004; Kolb et al. 2003). Therefore, thromboprophylaxis has been recommended in several international guidelines (Members et al. 2012; Jacobs et al. 2012; Falck-Ytter et al. 2012). In 2012, American College of Chest Physicians guidelines on antithrombotic and thrombolytic therapy were published (ACCP9), and it was recommended that anticoagulant should be used in patients undergoing hip fracture surgery for at least 10–14days postoperatively, but preferably for as much as 28–35days (Falck-Ytter et al. 2012). Several clinical studies and meta-analyses have shown that extended chemoprophylaxis significantly reduces the incidence of symptomatic DVT in orthopedic surgery patients (Fisher et al. 2013; Kakkar et al. 2008; QuinlanDaniel et al. 2002).

New oral anticoagulants are recommended, but LMWH are suggested as the preferred option for patients undergoing total hip arthroplasty (THA) or total knee arthroplasty (TKA) (Francis 2013). Some studies reported that new oral anticoagulants as rivaroxaban were safe and efficacy to reduce VTE rate in hip fracture patients (Long et al. 2014; Turpie et al. 2012). There is a clear trend toward shorter hospitalization of patients after major orthopedic surgery, leading to greater importance of outpatient thromboprophylaxis (Bergqvist et al. 2012). This means that the vast majority of patients may receive a daily pharmacological prophylaxis at home when discharge from hospital. However, the compliance may be a barrier of thromboprophylaxis, (Rahme et al. 2008) reported that fewer than 1 in 5 elderly patients discharged home after a hip or knee replacement surgery received postdischarge thromboprophylaxis. Few studies have assessed patient compliance with thromboprophylaxis after hospital discharge suggesting that patients can learn to self-administer treatments and comply with thromboprophylactic regimens (Colwell et al. 2005; Watts and Howie 2006; Mazor et al. 2007; Fisher and Turpie 2008; Spahn 2014). However, there is little information about the thromboprophylactic compliance in the real world, especially in Chinese population. As insufficient community health services were provided in China (Anand et al. 2008), we were concerned that the thromboprophylactic compliance would be poor. Thus the aim of this study was to assess the prevalence of thromboprophylaxis compliance and its impacts on the rate of VTE after surgery of hip fracture within 6weeks.

## Methods

### Study design and patients

We conducted a retrospective cohort study using admissions, physician claims, drug claims, demographic records and diagnosis information obtained from the electronic medical records of the General Hospital of PLA between January 2008 and December 2012. Eligible patients were those aged 50years or older who had femur neck fractures or intertrochanteric fractures. Exclusion criteria included: pathological fractures; did not undergo surgery for various reasons; diagnosed with any type of VTE at admission or before surgery; be contraindicated to pharmacological prophylaxis because of risk of bleeding. The study protocol was approved by the Ethic Committee of Chinese PLA General Hospital. Due to its retrospective nature and the fact that the patient data were anonymous, informed consent was not requested from patients.

### Thromboprophylaxis regimens and definition of compliance

All the hip fracture patients received routine drug and or mechanical preventive measures according to current guidelines. The drugs were used from the evening of the day the patients were admitted to hospital to the evening of the day before surgery if the surgery were not emergency. Subsequently, the regimen was resumed at least 8h after surgery and continued until discharge. The attending physicians would prescribe anticoagulants for every eligible patients and recommend a standard duration of 35days of chemoprophylaxis when discharged from hospital.

The pharmacological prophylaxis included unfractionated heparin (UFH), LMWH (nadroparin), fondaparinux, danaparoid and rivaroxaban. The LMWH was the first choice of chemoprophylaxis and followed by rivaroxaban and fondaparinux. Mechanical thromboprophylaxis was performed on all patients unless the patients refused to used it. The information of drugs used for thromboprophylaxis were collected from the prescriptions of every patient and an outpatient interview or telephone interview.

We defined thromboprophylaxis compliance as any type of pharmacological prophylaxis extended to 28days postoperatively (good compliant group, GC). Partial compliance was defined as prophylaxis continuing to 14–27days postoperatively (poor compliant group, PC), and non-compliance was defined as prophylaxis shorter than 14days or did not receive any pharmacological prophylaxis (non-compliant group, NC).

### Outcome measures

After admission to hospital, laboratory investigations such as routine blood tests, biochemistry and coagulation tests, were performed on every patients. And deep venous thrombosis was assessed by means of whole leg compression ultrasound (CUS) at admission. Whole leg CUS was repeated in patients who suffered from symptoms of suspected deep vein thrombosis (DVT) such as lower limb swelling, local tenderness and unexplained fever. A diagnosis of DVT was made based on the results of ultrasound. Lung ventilation/perfusion scanning or spiral CT was performed in patients with chest pain, breathing difficulties and other symptoms of suspected pulmonary embolism (PE). A diagnosis of PE was based on the results of the examinations. All patients returned to the clinic at 4, 8, 12weeks postoperatively and the would be recommended to received whole leg CUS examination if any symptoms of suspected DVT or PE were detected.

Major bleeding events was defined as bleeding that was fatal, that involved a critical organ, or that required reoperation or clinically overt bleeding outside the surgical site that was associated with a decrease in the hemoglobin level of 2g or more per deciliter or requiring infusion of 2 or more units of blood. Minor bleeding events including hemorrhagic wound complications such as excessive wound hematoma or bleeding at the surgical site.

The primary outcome of this study was the incidence of symptomatic DVT, PE within 6weeks postoperatively as the standard duration of chemoprophylaxis was 5weeks after hip fracture surgery.

### Statistical analysis

Student’s *t* test was used to analyze continuous data for comparisons of the baseline characteristics of the various patients groups. Pearson’s Chi square test, Fisher’s exact test and analysis of variance (ANOVA) were used for inter-group or inner-group comparisons of constituent ratios. Logistic regression analysis was used for 2-category data. Multivariate analysis was performed by the stepwise maximum likelihood method, and 95% confidence intervals (CIs) and odds ratio (OR) values were used to describe the hazard ratio. A P value less than 0.05 was considered statistically significant. All statistical procedures were accomplished with SPSS (SPSS Inc., IBM, USA, Version 19.0).

## Results

### Patient characteristics

**Fig.1:**
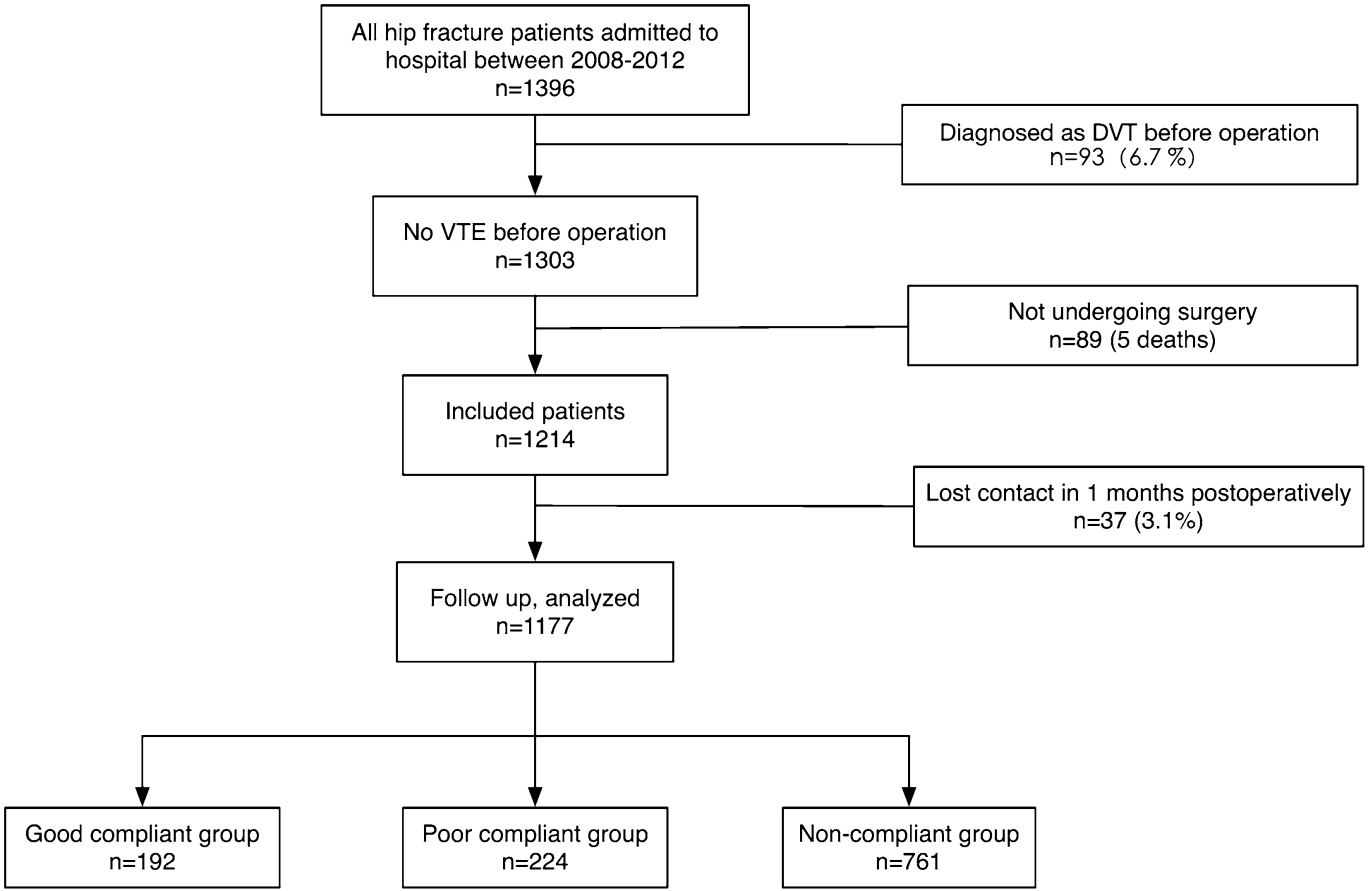
Flowchart of included patients and data analysis in this study

In total, 1396 elderly patients admitted to our hospital suffering hip fracture during 2008 to 2012. Of these patients, 93 (6.7%) were diagnosed as deep vein thrombosis (DVT) at admission and been treated with anticoagulants, 89 did not undergo surgery for various reasons (five patients died before operation). Then, 1214 patients were included in this study. There were 37 (3.1%) patients lost contact during 1months follow-up periods, so the remained 1177 patients were analyzed at last. All patients entered/enrolled in this study are accounted for in Fig.1.

On average, all the patients were aged 75.2 (standard deviation [SD] 10.2) years range from 50 to 109years old. Of these patients, 63.0% were female, 54.5% were femur neck fracture, 761 were classified to NC group, 224 were PC group, and 192 were GC group.

We compared the demographic characteristics between three groups and data was shown in Table1. There were no significant differences about the age, gender, comorbidities among three groups. However, the proportion of femur neck fracture and patients received chemoprophylaxis before surgery was lower in NC group than others (P=0.001). The prevalence of mechanical thromboprophylaxis was highest and the coverage of medical insurance was lowest in GC group (P=0.001). Although all patients were prescribed any one of anticoagulants after discharge in our hospital, but only 16.3% were completely compliant with physicians’ advices.

**Table1:** The basic and demographic characteristics between three groups

	Non-compliant group		Poor compliant group		Good compliant group		P value
	N	%/SD	N	%/SD	N	%/SD	
Number	761	64.7	224	19.0	192	16.3	NA
Age	75.1	10.2	76.0	9.6	75.0	11.0	0.476
Gender (female)	476	62.5	146	65.2	119	62.0	0.738
Type of fxs (femur neck fracture)	378	49.7	141	62.9	122	63.5	0.001
History of VTE	13	1.7	4	1.8	3	1.6	0.984
Hormone replacement therapy	2	0.3	1	0.4	3	1.6	0.147
Diabetes	155	20.4	47	21.0	44	22.9	0.740
Hypertension	347	45.6	111	49.6	89	46.4	0.580
Ischemic heart disease	136	17.9	46	20.5	34	17.7	0.643
Cerebrovascular disease	113	14.8	40	17.9	30	15.6	0.551
Malignant tumor	33	4.3	11	4.9	10	5.2	0.847
Mechanical thromboprophylaxis	91	12.0	37	16.5	54	28.1	0.001
Chemophrophylaxia before surgery	532	69.9	179	79.9	165	85.9	0.001
Medical insurance	463	60.8	147	65.6	64	33.3	0.001

*VTE* venous thromboembolism, *fxs* fractures, SD standard deviation

### VTE incidence

Patients in NC group had a proportionally higher rate of DVT (9.6 vs. 5.4 vs. 4.2%, P=0.013) postoperatively, mainly driven by higher rates of distal DVT (7.1 vs. 4.0 vs. 3.1%, P=0.048). No significant differences in rates for proximal DVT or PE were not found (Table 2). No fatal PE was found in this study. The DVT rates were not significant different during hospitalization prior to discharge (3.4 vs. 2.2 vs. 3.1%, P=0.671), however it was obviously higher in NC and PC group (6.2 vs. 3.1 vs. 1.0%, P=0.005).

**Table2:** VTE rates and bleeding events in all three groups

	Non-compliant group		Poor compliant group		Good compliant group		P value
	N	%	N	%	N	%	
Number	761		224		192		NA
PE in 1month	2	0.3	2	0.9	0	0.0	0.241
DVT in 1month	73	9.6	12	5.4	8	4.2	0.013
Proximal DVT	19	2.5	3	1.3	2	1.0	0.282
Distal DVT	54	7.1	9	4.0	6	3.1	0.048
VTE in hospital	26	3.4	5	2.2	6	3.1	0.671
VTE after discharge	47	6.2	7	3.1	2	1.0	0.005
VTE in total	73	9.6	12	5.4	8	4.2	0.001
Major bleeding events	4	0.5	3	1.3	1	0.5	0.474
Minor bleeding events	16	2.1	10	4.5	6	3.1	0.150

*VTE* venous thromboembolism, *DVT* deep venous thrombosis, *PE* pulmonary embolism, *SD* standard deviation

Patients all experienced low rates of major or minor bleeding events in three groups (Table 2), and it showed no significant difference (P=0.301, P=0.166 separately). No fatal bleeding events or bleeding into a critical site were found in this study, all patients experienced major bleeding events received effectively treatment.

### Multivariate analysis

In multivariate analysis, the history of VTE, HRT, malignant tumor and non compliance were confirmed to be independent risk factors of VTE after surgery of hip fracture (Table 3). The odds ratio (OR) of non-compliant compared with good compliant was 2.77 (95% confidence interval [CI], 1.27–6.04; P=0.010).

**Table3:** Univariate and multivariate analysis using logistic regression models to evaluate the potential risk factors for the occurrence of VTE in hip fracture patients

	P value	OR	95 % lower CI	95 % higher CI
Univariate analysis				
Age	0.851	1.002	0.981	1.023
Gender (female)	0.920	1.023	0.659	1.588
Type of fxs (femur neck fracture)	0.314	0.804	0.527	1.229
History of VTE	0.001	5.271	1.976	14.058
Hormone replacement therapy	0.003	12.011	2.390	60.372
Diabetes	0.518	0.835	0.484	1.441
Hypertension	0.486	0.859	0.560	1.317
Ischemic heart disease	0.258	0.705	0.385	1.292
Cerebrovascular disease	0.293	1.34	0.78	2.30
Malignant tumor	0.851	1.002	0.981	1.023
Mechanical thromboprophylaxis	0.629	1.149	0.654	2.018
Chemophrophylaxia before surgery	0.763	0.93	0.58	1.50
Medical insurance	0.211	1.32	0.85	2.05
Poor compliant	0.572	1.302	0.521	3.254
Non-compliant	0.019	2.440	1.155	5.155
Multivariate analysis				
History of VTE	0.001	5.58	2.05	15.21
Hormone replacement therapy	0.002	15.28	2.64	88.35
Malignant tumor	0.048	2.24	1.01	4.97
Poor compliant	0.476	1.41	0.55	3.63
Non-compliant	0.010	2.77	1.27	6.04

*VTE* venous thromboembolism, *fxs* fractures, *SD* standard deviation, *OR* odds ratio, *CI* confidence interval

## Discussion

We were not surprised about such a low rate of compliance of thromboprophylaxis in China. We found an extremely low compliant rate in our hip fracture patients after surgery. There were only 16.6% hip fracture patients adhered to the doctors’ prescription after discharge. This finding is in line with published studies no matter which drugs been used (Rahme et al. 2008; Colwell et al. 2005; Watts and Howie 2006; Fisher and Turpie 2008; Spahn 2014; Karlinski et al. 2006). Adamali et al. (2013) established a national house-staff audit to assess the compliance of thromboprophylaxis in medical patients and found the compliance rate was lower than 29.7%. This is unacceptable according to the ACCP guidelines and required to improve the compliance. If patients are non-compliant after discharge, they may miss approximately 40–50% of their entire thromboprophylaxis (Wilke et al. 2010). The overwhelmed researches show that clinically relevant DVT and PE occur at a median of 20 and 21days after TKA and 12 and 34days after THA (Warwick et al. 2007; Bjornara et al. 2006). Therefor, non-compliance may had detrimental resistance on the thromboprophylaxis efficiency.

We afterwards confirmed that the non-compliance was correlated with higher VTE rate. This situation is very depressed for both doctors and patients. Even when we adjusted with other risk factors of VTE, such as age, gender, fracture type, mechanical thromboprophylaxis and chemoprophylaxis before surgery, the compliance of thromboprophylaxis was independently associated with higher VTE incidence postoperatively. The extended duration of chemoprophylaxis is significantly more effective in preventing venous thromboembolism in orthopedic surgery patients than the recommended practice of at least 10days. Our results demonstrated that extended duration of more than 4weeks was significantly more effective than less than 2weeks. The compliance of thromboprophylaxis after discharge was the key stone of preventing venous thromboembolism. We should noticed that, although the VTE rate was significantly higher in GC group than NC group, it was not significant different between GC group and PC group. The multivariate analysis confirmed that non-compliant was an independent risk factor for VTE (OR 2.77, 95% CI 1.27–6.04), however, the poor compliant was not independent (OR 1.41, 95% CI 0.55–3.63). This means that we should ensure 2weeks of chemoprophylaxis at least after hip fracture surgery to preventing venous thromboembolism effectively.

Although more patients in GC group received chemoprophylaxis before surgery, it could not affect the postoperative VTE rate as the patients diagnosed VTE were excluded from this study. And the multivariate analysis confirmed its influence was limited. Considering the different proportion of mechanical thromboprophylaxis in different groups, we compared the VTE rate during hospitalization prior to discharge. The results revealed that no significant differences were detected during this period, when the mechanical devices were applied.

In our study, we were able to show the existence of high rate of non-compliance in hip fracture patients after discharge. Reasons for this were very complex. Objectively, the lack of primary healthcare institutions and deficiency of home nursing in China were main causes of non-compliance (Gong 2012). The drug-related problems may be main reason for such a high non-compliance rate in this study as we do not have a comprehensive network of inpatients rehabilitation clinics in China. This enforced our patients to inject anticoagulants at their home and this is very hard to apply. Additionally, insurance was another factor influencing the compliance of patients. Insurance coverage may influenced the compliance after discharge because the new oral anticoagulants were not covered by medical insurance right now. We observed that self-paying patients complied with doctors’ advices and preferred to pay for the anticoagulants. So the proportion of insurance was rather low in compliance group. Subjectively, the health literacy of Chinese adults should be improved as the poor public understanding of importance of thromboprophylaxis may be great barriers. And also the communication between doctors and patients usually was insufficient in our country.

Our results suggested an urgent improvement on the thromboprophylaxis compliance after hip fracture surgery. The patients’ education regarding thrombosis risk may be effective to improve the compliance (Mazor et al. 2007). The SALTO study found that adherence to and satisfaction with the oral thromboprophylaxis were better than for drop by injection in the context of outpatient’s prolongation (Peidro-Garces et al. 2013). Several studies demonstrated that the prescription of rivaroxaban could afford a superior patient compliance compared with subcutaneous LMWH or enoxaparin (Carrothers et al. 2014; Rubenacker et al. 2013; Rogers et al. 2010). And Wilke’s survey revealed the patients appeared to have a positive preference for oral thromboprophylaxis after major orthopedic surgery (Wilke 2009).

## Limitations

There were some limitations in our study. Firstly, due to the retrospective design, recall bias was unavoidable and we did not contain the medical history of warfarin, aspirin and NSAIDs. For example, patients may have misremembered the anticoagulant they used and duration of prophylaxis. The diagnosis of VTE was based on patient’s symptoms and physician’s judgment, thus may underestimated the VTE rate. Additionally, the definition of compliance was established on the extension to 35days thromboprophylaxis as recommended by ACCP9 guideline. However, we could not treat all patients as same situation because some patients were received individual advices by their doctors.

## Conclusions

In this study we found fewer than 1 in 5 patients maintained compliance in thromboprophylaxis after discharge following hip surgery despite clinical guidelines. This is particularly concerning because those who were non-compliance had higher risk of VTE within 3months postoperatively compared with those who were partial or completely compliance in thromboprophylaxis.

## References

[CR1] Adamali H (2013). A national house-staff audit of medical prophylaxis in medical patients for the prevention of venous thromboembolism (prevent-vte). Ir Med J.

[CR2] Anand S (2008). China’s human resources for health: quantity, quality, and distribution. Lancet.

[CR3] Anderson FA (2002). Prolonged prophylaxis in orthopedic surgery: insights from the USA. Semin Thromb Hemost.

[CR4] Bergqvist D (2012). Evaluation of the duration of thromboembolic prophylaxis after high-risk orthopaedic surgery: the ETHOS observational study. Thromb Haemost.

[CR5] Bjornara BT, Gudmundsen TE, Dahl OE (2006). Frequency and timing of clinical venous thromboembolism after major joint surgery. J Bone Joint Surg Br.

[CR6] Carrothers AD (2014). Patient-reported compliance with thromboprophylaxis using an oral factor xa inhibitor (rivaroxaban) following total hip and total knee arthroplasty. J Arthroplasty.

[CR7] Colwell CW (2005). Patient compliance with outpatient prophylaxis: an observational study. Orthopedics.

[CR8] Falck-Ytter Y (2012). Prevention of VTE in orthopedic surgery patients: antithrombotic therapy and prevention of thrombosis, 9th ed: American college of chest physicians evidence-based clinical practice guidelines. Chest.

[CR9] Fisher WD, Turpie AGG (2008). Outpatient thromboprophylaxis after hip or knee surgery: discrepancies and concerns. Can Med Assoc J.

[CR10] Fisher WD (2013). Extended venous thromboembolism prophylaxis in patients undergoing hip fracture surgery—the SAVE-HIP3 study. Bone Joint J.

[CR11] Francis CW (2013). Prevention of VTE in patients having major orthopedic surgery. J Thromb Thrombolysis.

[CR12] Geerts WH (2008). Prevention of venous thromboembolism. Chest.

[CR13] Goldhaber SZ (2012). Venous thromboembolism: epidemiology and magnitude of the problem. Best Pract Res Clin Haematol.

[CR14] Gong P (2012). What can be learned from China’s health system?. Lancet.

[CR15] Haentjens P, De Groote K, Annemans L (2004). Prolonged enoxaparin therapy to prevent venous thromboembolism after primary hip or knee replacement. A cost-utility analysis. Arch Orthop Trauma Surg.

[CR16] Jacobs JJ (2012). American Academy of Orthopaedic Surgeons clinical practice guideline on: preventing venous thromboembolic disease in patients undergoing elective hip and knee arthroplasty. J Bone Joint Surg Am.

[CR17] Kakkar AK (2008). Extended duration rivaroxaban versus short-term enoxaparin for the prevention of venous thromboembolism after total hip arthroplasty: a double-blind, randomised controlled trial. Lancet.

[CR18] Karlinski M (2006). Compliance with low molecular weight heparin in abulatory orthopedic patients. Ortop Traumatol Rehabil.

[CR19] Kolb G (2003). Reduction of venous thromboembolism following prolonged prophylaxis with the low molecular weight heparin Certoparin after endoprothetic joint replacement or osteosynthesis of the lower limb in elderly patients. Thromb Haemost.

[CR20] Long A (2014). Efficacy and safety of rivaroxaban versus low-molecular-weight heparin therapy in patients with lower limb fractures. J Thromb Thrombolysis.

[CR21] Mazor KM (2007). Patient education about anticoagulant medication: is narrative evidence or statistical evidence more effective?. Patient Educ Couns.

[CR22] Members o (2012). Preventing venous thromboembolic disease in patients undergoing elective total hip and knee arthroplasty. J Bone Joint Surg Am.

[CR23] Peidro-Garces L, Otero-Fernandez R, Lozano-Lizarraga L (2013). Adherence to and satisfaction with oral outpatient thromboembolism prophylaxis compared to parenteral: SALTO study. Rev Esp Cir Ortop Traumatol.

[CR24] Prandoni P (2002). Prolonged thromboprophylaxis with oral anticoagulants after total hip arthroplasty: a prospective controlled randomized study. Arch Intern Med.

[CR25] QuinlanDaniel J, Eikelboom John W, Douketis James D (2002). Anticoagulants (extended duration) for prevention of venous thromboembolism following total hip or knee replacement or hip fracture repair. Cochrane Database Syst Rev.

[CR26] Rahme E (2008). Postdischarge thromboprophylaxis and mortality risk after hip-or knee-replacement surgery. CMAJ.

[CR27] Rogers BA (2010). Is there adequate provision of venous thromboembolism prophylaxis following hip arthroplasty? An audit and international survey. Ann R Coll Surg Engl.

[CR28] Rubenacker S, Kaiser J, Guschmann M (2013). Compliance of patients undergoing thromboprophylaxis with enoxaparin: the COMFORT study. Chirurg.

[CR29] Spahn G (2014). Compliance with self-administration of heparin injections in outpatients. Eur J Trauma.

[CR30] Turpie AGG (2012). XAMOS: a non-interventional study comparing oral rivaroxaban with conventional regimens for thromboprophylaxis after major orthopaedic surgery of the hip and knee. Br J Haematol.

[CR31] Warwick D (2007). Insufficient duration of venous thromboembolism prophylaxis after total hip or knee replacement when compared with the time course of thromboembolic events: findings from the global orthopaedic registry. J Bone Joint Surg Br.

[CR32] Watts AC, Howie CR (2006). Assessment of a self-administration protocol for extended subcutaneous thromboprophylaxis in lower limb arthroplasty. J Bone Joint Surg Br.

[CR33] Wilke T (2009). Patient preferences for an oral anticoagulant after major orthopedic surgery: results of a german survey. Patient.

[CR34] Wilke T (2010). Nonadherence in outpatient thrombosis prophylaxis with low molecular weight heparins after major orthopaedic surgery. Clin Orthop Relat Res®.

